# Recent Advances on Pathophysiology, Diagnostic and Therapeutic Insights in Cardiac Dysfunction Induced by Antineoplastic Drugs

**DOI:** 10.1155/2015/138148

**Published:** 2015-10-25

**Authors:** Marilisa Molinaro, Pietro Ameri, Giancarlo Marone, Mario Petretta, Pasquale Abete, Fabio Di Lisa, Sabino De Placido, Domenico Bonaduce, Carlo G. Tocchetti

**Affiliations:** ^1^Department of Medicine and Health Sciences, University of Molise, 86100 Campobasso, Italy; ^2^Department of Internal Medicine, University of Genova, 16132 Genova, Italy; ^3^Department of Clinical Medicine and Surgery, Federico II University, 80131 Naples, Italy; ^4^Department of Translational Medical Sciences, Division of Internal Medicine, Federico II University, 80131 Naples, Italy; ^5^Department of Biomedical Sciences, University of Padova, 35121 Padova, Italy; ^6^National Researches Council, Neuroscience Institute, University of Padova, 35121 Padova, Italy

## Abstract

Along with the improvement of survival after cancer, cardiotoxicity due to antineoplastic treatments has emerged as a clinically relevant problem. Potential cardiovascular toxicities due to anticancer agents include QT prolongation and arrhythmias, myocardial ischemia and infarction, hypertension and/or thromboembolism, left ventricular (LV) dysfunction, and heart failure (HF). The latter is variable in severity, may be reversible or irreversible, and can occur soon after or as a delayed consequence of anticancer treatments. In the last decade recent advances have emerged in clinical and pathophysiological aspects of LV dysfunction induced by the most widely used anticancer drugs. In particular, early, sensitive markers of cardiac dysfunction that can predict this form of cardiomyopathy before ejection fraction (EF) is reduced are becoming increasingly important, along with novel therapeutic and cardioprotective strategies, in the attempt of protecting cardiooncologic patients from the development of congestive heart failure.

## 1. Introduction

The prognosis of cancer has dramatically improved in the last decades: several types of malignancies can be now cured or maintained in remission for a long time and patients can live the remainder of their lives free of disease. However, they are also exposed to chronic complications of antineoplastic treatments. Many classes of chemotherapeutic drugs can impair cardiovascular homeostasis and favor or even trigger cardiovascular disorders. The more the survival of oncological patients increases, the higher is the likelihood that cardiovascular consequences of cancer therapies become the major health problem after tumor elimination is achieved. The most common side effects of anticancer treatment include vasospastic and thromboembolic ischemia, arterial hypertension, arrhythmia, and cardiac dysfunction up to heart failure (HF) [1, 2]. The latter is an especially fearful long-term complication of chemotherapy because it remains a slowly progressing condition that ultimately can only be resolved by heart transplantation. Nevertheless, this procedure can be offered only to a small percentage of subjects due to the limited availability of donor organs. In fact, the number of heart transplants has remained static worldwide and the number of heart transplants performed each year in the United States has plateaued at about 2100 for the past few years (2001 Heart and stroke statistical update. Dallas: American Heart Association, 2000).

Here we first give an updated overview of the main characteristics and mechanisms of chemotherapy-associated cardiac toxicity, since a thorough knowledge of this phenomenon can provide important hints to predict, treat, and prevent it. Special attention is paid for chemotherapy-related cardiac dysfunction, in the light of the clinical and social burden of heart failure that may ensue [3, 4]. Next, we examine the approaches that have already been implemented in clinical practice or are currently being investigated for the prompt diagnosis and effective management of chemotherapy cardiotoxicity.

## 2. Classification of Chemotherapy-Related Cardiotoxicity

Left ventricular (LV) dysfunction induced by* anthracyclines* has historically been the most relevant form of chemotherapy cardiotoxicity [7]. Nevertheless, new oncological drugs, such as intracellular signaling inhibitors, may be also cardiotoxic, as they target pathways that also play a major role in the maintenance of cardiac homeostasis, especially when during stressful conditions, such as hypertension or hypertrophy [1]. For instance,* human epidermal growth factor receptor 2 (HER/ErbB2)* and* angiogenesis inhibitors*, which have entered clinical practice in relatively recent years, profoundly affect cardiac metabolism and contractile proteins (for important reviews on such mechanisms, please refer to [2, 8–12]). This type of toxicity does not display cardiomyocyte disruption, is most often reversible with treatment discontinuation, and has been named type II LV dysfunction [13]. Conversely, cardiotoxicity produced by anthracyclines is typically irreversible, with marked ultrastructural myocardial derangements, and is referred to as type I [13]. However, these two paradigms of cardiotoxicity may overlap: for example, the anti-ErbB2 antibody,* trastuzumab*, can trigger irreversible cardiac damage in patients previously treated with anthracyclines [14].

## 3. Cardiotoxicity of Anthracyclines

*Anthracyclines* are antibiotics belonging to the family of rodomicine, originally isolated from* Streptomyces peucetius*, with very potent antineoplastic activity [15]. In particular,* doxorubicin* and* epirubicin* are currently the cornerstone of treatment of many malignancies, including breast cancer, lymphomas, and sarcomas. It has been estimated that approximately 10% of patients receiving doxorubicin or its derivatives will develop cardiac complications, even up to 10 years after the completion of chemotherapy [1]. However, endomyocardial biopsy studies and seriate measurements of troponin I have revealed that cardiac cell alterations already occur during or a few hours after exposure to anthracyclines, regardless of when clinical manifestations appear. Furthermore, an early and subclinical deterioration of systolic function can be detected in most patients exposed to anthracyclines with Tissue Doppler or Speckle Tracking echocardiography [16, 17]. The delay between cardiac injury and clinical presentation may be explained by the fact that anthracycline cardiotoxicity is temporarily compensated for by the activation of protective signaling pathways and by a myocardial functional reserve [18, 19].

The probability of developing anthracycline cardiomyopathy is primarily dose dependent [20]. Additional risk factors are genetic predisposition, very young or old age, female gender, intravenous bolus infusion, hypertension, diabetes mellitus, preexisting cardiac disease, previous or concurrent mediastinal radiation therapy, and combination with alkylating or antimicrotubule chemotherapeutics [1, 21–26]. Thus, accurate medical history may be helpful in identifying individuals susceptible to anthracycline cardiotoxicity. However, it should be noted that many of the aforementioned risk factors have been identified over relatively short follow-up periods and that long-term investigations are needed to confirm their relevance [1].

### 3.1. Molecular Mechanisms of Anthracycline Cardiotoxicity

**Figure 1:**
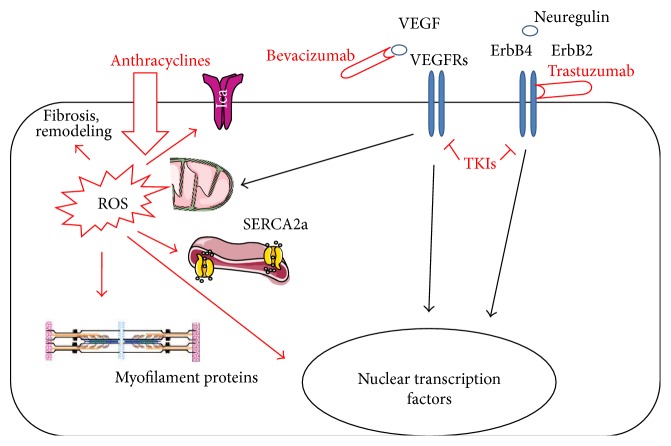
Schematic representation of the main mechanisms by which cardiomyocytes are damaged by the most cardiotoxic anticancer agents among those currently in use. Anthracyclines induce a DNA damage response and reactive oxygen species (ROS) production; these two initial events result in a cascade of secondary alterations affecting mitochondrial integrity and function, intracellular calcium dynamics, and contractile proteins. By blocking the activity of tyrosine kinase receptors, such as vascular endothelial growth factor receptor (VEGFR) or ErbB2/ErbB4, bevacizumab, trastuzumab, and tyrosine kinase inhibitors (TKIs) alter mitochondria and modulate gene expression. SERCA2a: sarcoendoplasmic reticulum calcium ATPase. Black arrows indicate physiologic, homeostatic effects. Red arrows indicate deleterious effects. Modified from [5, 6].

*Anthracyclines* are DNA intercalating agents that form a ternary complex with topoisomerase 2. This enzyme transiently breaks the DNA backbone to untangle the supercoiled DNA complex in a process required for transcription, replication, and recombination [2, 27, 28]. Under physiological conditions topoisomerase 2 reanneals the cut strands. Conversely, when the complex with anthracyclines is formed, the relegation is inhibited resulting in an uncontrolled occurrence of DNA strand breaks. The resulting cascade of molecular events, referred to as DNA damage response, eventually leads to mitochondrial dysfunction and accumulation of reactive oxygen species (Figure 1) [27]. Consistent with this model,* doxorubicin* cardiotoxicity is prevented in mice knockout for the gene encoding the cardiac isoform of topoisomerase 2 [27]. Besides eliciting the DNA damage response, anthracyclines also cause the formation of reactive oxygen species by accepting and immediately releasing electrons onto the oxygen molecules present inside the cardiomyocyte, especially in mitochondria [15, 27–31]. Furthermore, anthracyclines induce the intracellular accumulation of iron and form complexes with it, further inducing the production of free oxygen radicals via metal-catalyzed oxidoreductions [15, 29–31]. The DNA damage response and oxidative stress initiate a number of secondary cellular alterations, such as changes in calcium homeostasis and abnormalities of the contractile apparatus [15, 29–31]. At the ultrastructural level loss of myofibrils, dilation of the sarcoplasmic reticulum and cytoplasmic vacuolization are observed [15, 29–31]. Eventually, cardiomyocytes may die or undergo senescence following exposure to anthracyclines [32]. This can be because of direct toxicity of anthracyclines or as a result of the impairment of antiapoptotic signaling axis. For instance, our recent work has pinpointed a state of resistance to insulin-like growth factor-1, a hormone fundamental for cardiomyocyte survival, as a mechanism of doxorubicin-triggered death of cardiac cells [33, 34]. It has been proposed that apoptosis and senescence of cardiac progenitor cells chiefly contribute to the pathogenesis of anthracycline cardiomyopathy, as depletion of these cell population hinders the ability of the heart to regenerate in response to minor injuries which, thereby, accumulate and affect cardiac structure and function [35, 36].

Moreover, it is conceivable that anthracyclines also alter the activity of cardiac fibroblasts and the turnover of the myocardial extracellular matrix. Doxorubicin enhances the expression of MMP2 and MMP9, thus weakening the collagenous matrix and contributing to myocardial remodeling [15, 37, 38]. Indeed, fibrosis is observed in hearts that have been exposed to doxorubicin [36] and may impinge on both diastolic and—via misalignment of cardiomyocytes—systolic function.

Anthracyclines also induce a local immune response, with the involvement of dendritic cells and distinct subsets of T lymphocytes, which may underlie part of the antineoplastic effect [39]. However, immune activation and inflammation may be harmful to the heart. Since anthracycline-triggered inflammation is at least in part secondary to the activity of IL-1*β*, suppression of the latter might blunt some of the adverse inflammatory effects that complicate chemotherapy with anthracyclines [40].

### 3.2. Other Agents

*Mitoxantrone* is an anthracycline analog that can damage myocytes, resulting in LV dysfunction similarly to anthracycline [1, 41]. Large single doses of* cylophosphamide* are able to cause hemorrhagic cell necrosis, bringing to heart failure or even death. Such toxic effects are seen very rarely since lower doses are being used these days [1, 42]. Another drug that has been linked to late-onset LV dysfunction (milder than anthracyclines) is* cisplatin* [1, 43].

Also,* taxanes* such as* paclitaxel* and* docetaxel* are antimicrotubule agents that bind to tubulin, thus impairing the disassembly of microtubules and inhibiting cell division. They are widely used in the treatment of multiple malignancies. The incidence of HF associated with such drugs, according to retrospective analysis, is relatively low (1.6% among patients treated with docetaxel-doxorubicin-cyclophosphamide and 0.7% for those treated with 5-fluorouracil-doxorubicin-cyclophosphamide) [44, 45].

The antimetabolite* 5-fluorouracil (5-FU)* has been shown to cause angina-like chest pain and, in rare cases, myocardial infarction, arrhythmias, LV dysfunction, and sudden death [46–48]. In animal models, direct toxicity on the myocardium has been postulated. This could be due to myocardial accumulation of citrate that has been attributed to generation of fluoroacetate (formed from the degradation of 5-FU parenteral preparation) and can interfere with the Krebs cycle [48–51]. Also 5-FU can induce dose- and time-dependent depletion of high energy phosphates, apoptosis [48, 51–53], autophagy, ROS elevation, and senescence of cardiomyocytes and endothelial cells [54].

## 4. Cardiotoxicity of Type II Agents

### 4.1. Anti-ErbB2 Agents

The first and most widely used type II cardiotoxic drug is* trastuzumab*, a humanized monoclonal antibody against the extracellular domain IV of HER/ErbB2 [8, 9].

ErbB2 (also called HER2) is a member of the epidermal growth factor receptor family. Upon ligand binding, these transmembrane receptors homo- or heterodimerize, undergo transphosphorylation, and initiate a number of cellular responses. As no specific ligand for ErbB2 has been identified so far, it is believed that it normally functions as a dimerization partner of the other ErbBs [9]. By contrast, ErbB2 is overexpressed in about 30% of breast cancers, in which it spontaneously interacts with the other ErbBs independent of ligand stimulation, and triggers signaling cascade promoting tumor growth and survival [55]. Trastuzumab is highly effective in treating ErbB2-positive breast and also gastric cancers. However, it also causes cardiac dysfunction in a substantial proportion of patients, which was found to peak to 28% when trastuzumab is coadministered with anthracyclines [56, 57]. In fact, this association is now avoided.

As a class II cardiac dysfunction [58], trastuzumab-induced cardiac dysfunction appears to arise from impairment of contractility rather than loss of myocytes, and the release of troponin shown in sequential treatment with anthracyclines + trastuzumab seems to be ascribed to the previous chemotherapy [59]. EF is likely to recover and there is evidence that it is relatively safe to readminister trastuzumab after it has been discontinued and myocardial function has returned to baseline [13].

*Pertuzumab* is another, more recent anti-HER2 antibody that binds to the domain II of the receptor. A third HER2-targeting agent is* lapatinib*, a small molecule inhibitor of the intracellular tyrosine kinase domain of HER2. Trastuzumab only disrupts ligand-independent HER2 signaling; conversely, pertuzumab interferes with the formation of ligand-induced HER2 heterodimers. Lapatinib affects both ligand-triggered and ligand-independent HER2 signaling [9]. Interestingly, lapatinib seems to be less toxic than trastuzumab. Data about the toxicity of pertuzumab are limited [57].

Cardiotoxicity of HER2-targeting drugs has been ascribed to the inhibition of fundamental actions of neuregulin-1 in the heart [57, 60]. Neuregulin-1 acts on cardiac cells via ErbB4/ErbB4 homodimers and ErbB4/ErbB2 heterodimers to elicit protective pathways in response to stress (Figure 1) [60]. By blocking neuregulin-1 effects in the heart, HER2 inhibitors may make it more vulnerable to noxious stimuli, among which anthracyclines. Consistent with this interpretation, mice with cardiac-specific deletion of ErbB2 show dilated cardiomyopathy, with increased susceptibility to cardiomyocyte death after anthracyclines [61]. The ErbB2 pathway is required for cell survival and continuing function and seems to be activated when the myocardium faces adverse hemodynamics or other stress, such as anthracycline therapies [62]. Upon withdrawal of trastuzumab, the normal ErbB2 pathway is reestablished, and the declined EF can return to normal, opposite to anthracyclines that produce a type I toxicity with permanent myocyte dysfunction. This is consistent with the increase in cardiotoxicity when trastuzumab is associated with anthracyclines: trastuzumab enhances or even uncovers the damage caused by anthracyclines. Once ErbB2 inhibitors block the ErbB2-triggered repair mechanisms, the oxidative damage induced by anthracyclines proceeds without control [59]. Indeed, experimental studies have shown that neuregulin 1 modulates doxorubicin damage in rat cardiomyocytes [14, 57, 63, 64].

With its cardioprotective features, neuregulin is now being intensively studied in clinical trials as a therapeutic for heart failure [65].

### 4.2. Antiangiogenic Drugs

Among drugs that induce type II cardiotoxicity we have to acknowledge antiangiogenic drugs. In particular,* bevacizumab*,* sorafenib*, and* sunitinib* are now widely used in oncology; more recently,* pazopanib* and* vandetanib* have also been approved by the US Food and Drug Administration [1, 66, 67]. All these drugs interfere with vascular endothelial growth factor (VEGF) signaling (Figure 1). As VEGF contributes to cardiomyocyte function and growth on the one hand and to the integrity and expansion of the coronary and systemic circulation on the other one [8, 10, 11, 45, 67–70], it is not surprising that VEGF antagonism may lead to cardiovascular side effects, principally hypertension, thromboembolism, LV dysfunction, and HF [71–73]. Indeed, like cancer, the heart is highly dependent on adequate perfusion for its normal function [8, 10, 11, 45, 67–70], both relying on similar HIF-1 and VEGF pathways. Indeed, the inhibition of HIF-1 by p53 causes cardiac dysfunction during chronic pressure overload [74], and conditional expression of a VEGF scavenger caused microvessel rarefaction and myocardial hibernation which was fully reversible even months after switching off the expression of the scavenger [75, 76]. These data suggest that the heart is especially sensitive to antiangiogenic therapies in the setting of hypertension-related pressure overload.

Bevacizumab is an antibody, which binds specifically to circulating VEGF-A (that activates signaling in endothelial cells), and is currently approved for the treatment of advanced carcinoma of the lung, breast, and colon-rectum [77, 78]. Bevacizumab has been reported to induce LV dysfunction in 1% of chemotherapy-naïve patients and 3% of patients who have already received chemotherapy [79]. Instead, sunitinib and sorafenib, which are used in metastatic renal cancer and in imatinib-resistant gastrointestinal stromal tumors [72, 80], belong to the class of small molecule tyrosine kinase inhibitors. They are not very selective and also block signaling cascades other than the one of VEGF [10]. In particular, sunitinib inhibits more than 30 other receptor and nonreceptor tyrosine kinases, including c-Kit, platelet-derived growth factor receptor (PDGFR) alpha and beta, rearranged during transfection (RET), FMS-related tyrosine kinase 3 (FLT3), and colony-stimulating factor 1 receptor (CSF1R) [8, 10, 39, 81], which may be why it appears to be more cardiotoxic than other angiogenesis inhibitors, with a reported decrease in EF in up to 28% of treated patients [82–85]. Seminal studies [86–90] have proven the importance of these pathways in cardiovascular homeostasis. The higher incidence of sunitinib cardiotoxicity is also explained by inhibition of off-target kinases, such as ribosomal S6 kinase (RSK), with consequent activation of the intrinsic apoptotic pathway, and 5′ AMP-activated protein kinase (AMPK, important for the response to energy stress), with worsening of ATP depletion [8, 91]. Therefore, LV dysfunction would occur due to myocyte dysfunction. In mice treated with sunitinib and exposed to pressure load, Chu and colleagues [82] observed that cardiomyocytes exhibited opening of the mitochondrial permeability transition pore and marked mitochondrial swelling with destruction of the normal mitochondrial architecture. Moreover, direct administration of sunitinib on different myocardial preparations results in a dose-dependent inotropic effect, accompanied by decline in intracellular Ca^2+^ and increased reactive oxygen species (ROS) production [67, 92].

At clinically relevant concentrations in* in vitro* kinase assay, sorafenib inhibits at least 15 kinases, including VEGF receptor, PDGFR, Raf-1/B-Raf, c-Kit, and FLT3 [8, 10, 67]. The rate of cardiotoxicity associated with sorafenib is not yet clear. Two meta-analysis, including almost 7000 patients treated with sunitinib and 900 patients treated with sorafenib, found a 4.1% rate of sunitinib-induced HF and 1% for sorafenib-associated cardiac dysfunction [93, 94], but most of these data are from retrospective analyses; only few trials have evaluated cardiac function and HF prospectively. Schmidinger and colleagues [71] reported that 3 out of 14 patients treated with sorafenib who experienced cardiac events showed abnormal EF.

Interestingly, a recent work from the Paolocci group [95] reported that a tyrosine kinase-receptor such as TrkB, with its endogenous ligand BDNF, is able to modulate the cardiac excitation-contraction coupling process directly, independently and in parallel to G protein-coupled receptor signaling. Such findings corroborate the concept that tyrosine kinase inhibition during anticancer therapies can disrupt important signaling, leading to consequent derangements in cardiac mechanical work that may largely contribute to loss in LV function [96].

Significant hypertension is seen with all three major antiangiogenic agents [97]. Bevacizumab results in a more serious form of hypertension that, at least in some instances, does not reverse with the removal of the offending agent. Remarkably, it has been suggested that drug-induced hypertension may be a biomarker of anticancer efficacy since patients who developed hypertension survived longer than those who did not [98]. In the work of Scartozzi and colleagues [99] on metastatic colorectal cancer patients, 20% of patients developed grade 2-3 hypertension. A partial remission was observed in 75 % of patients with bevacizumab-related hypertension and only in 32% of those without hypertension. Furthermore, patients who developed grade 2-3 hypertension had significantly longer progression-free survival than nonhypertensive patients [99].

### 4.3. Other Type 2 Agents

The BCR-ABL inhibitors,* imatinib* and* dasatinib*, are tyrosine kinase inhibitors used for treatment of chronic myelogenous leukemia and gastrointestinal stromal tumors. These two drugs were initially reported to induce HF, but large follow-up studies did not confirm such data [1, 100, 101].

## 5. Assessment and Treatment of Cardiac Damage during Cancer Treatment

Assessment of anticancer drug-related cardiotoxicity is an essential procedure before, during, and after treatment with these drugs. The majority of currently used methods used to assess cardiac function cannot differentiate between irreversible and reversible cardiotoxicity and may mislead physicians to stop potentially lifesaving cancer therapies. Cardiovascular side effects such as myocardial ischaemia, arterial hypertension, and dysrhythmia can be readily diagnosed, but detection of cardiac dysfunction is more challenging [1].

Preclinical screening for cardiotoxicity is fundamental for kinase inhibitors. Much preclinical screening focuses on the hERG (K^+^ channel) assay because many drugs increase risk of arrhythmia. Primary cell cultures of human cardiomyocytes dedifferentiate and die quickly over time; therefore, they are not a good reflection of what happens* in vivo*. In the future, stem-cell-based assays and assays based on the use of engineered heart tissue could be used. These assays could integrate effects on membrane action potential, calcium handling, myofilament function, gene expression, and cell survival [1, 8, 9, 11, 66, 67, 103].

For initial screening and detection of cardiac dysfunction in oncologic patients, along with ECG and physical assessment, noninvasive imaging with echocardiography or MUGA (Multiple Gated Acquisition) scans are now commonly used in cancer patients [1, 45, 57, 104, 105]. These methods are useful for evaluating patients for cardiotoxicity but have limited accuracy for risk stratification [1]. Attention should be paid not only to systolic but also to diastolic cardiac function. It should be noted that patients with advanced cancer may already have cardiovascular abnormalities such as fatigue, dyspnea, malaise, and propensity to severe arrhythmia. Distinguishing these from side effects attributable to cancer therapies requires a specific expertise.

One important and very active field of research is the search of new indexes of cardiac function other than the ejection fraction [106, 107]. Although strong outcomes data support MUGA for estimation of LVEF, such methodology is limited by radiation exposure. On the other hand, echocardiographic EF measurement is to be preferred for its simplicity and availability, but has the downside of being variable and insensitive [108]. Indeed, the normal heart has a huge recruitable contractile potential; therefore it must have undergone a considerable damage and myocyte loss in order for EF to be decreased [109]. On such basis, it is important to use other markers for cardiac function in the diagnostic armamentarium [57, 59, 105, 109–116]. More sensitive techniques to be used in the cardiotoxicity settings could be contrast that increases border definitions, enhancing accuracy and limiting interobserver variability [117–119], while echo-stress could evidence undiagnosed functional changes [119–121]. Tissue Doppler and strain techniques have been shown to detect anthracycline-induced cardiac dysfunction earlier than conventional echocardiography, but it is not known if these methods have a higher specificity to detect type I cardiotoxicity [122]. Instead, other superior imaging methodologies such as cardiac magnetic resonance (CMR) look promising (Table 1). A downside of this methodology is its limited availability, but it can provide improved accuracy and reproducibility of EF measurements [108]. Also, it has the unique property of characterizing the myocardial tissue, identifying myocardial inflammation, edema, and strain [119]. Other explored modalities include the use of the uptake of iodine-123-metaiodobenzylguanidine (MIBG), a radiolabeled analogue of norepinephrine, which decreases following cardiac damage [119, 123, 124]. Additionally, actomyosin antibodies could be used to detect myosin exposed after myocardial injury [119, 125, 126]. Finally, a predictor of cardiotoxicity may also be the uptake of radiolabeled chemotherapeutics [119, 127, 128].

The use of cardiac biomarkers (Table 1) can solve the limitations of cardiac imaging to stratify the risk in cancer patients with cardiac dysfunction. Cardiac biomarkers such as troponins and natriuretic peptides may be expected to be elevated with significant cardiotoxicity. Patients treated with anthracyclines showed a transient increase in brain natriuretic peptide (BNP), but the predictive value for long-term cardiotoxicity may be limited when such marker is used alone [129, 130]. Instead, troponins I and T have been shown to predict late anthracycline cardiotoxicity in children [131], and in an adult population they can identify anthracyclines-treated patients that can benefit from ACE-inhibitors [132]. In spite of these promising results, the assessment of cardiac biomarkers is not being performed routinely in patients undergoing cancer treatment, and multicentre trials to evaluate the role of biomarkers in this population are a need [1]. A 2014 study from Ky et al., while confirming TnI to be associated with LV dysfunction in patients with breast cancer undergoing sequential therapy with doxorubicin and trastuzumab, also showed that a marker of oxidative stress such as myeloperoxidase (MPO) could be mechanistically relevant to cardiotoxicity with cancer therapy [133].

All things said, there is no current established algorithm for preoncologic treatments evaluation and follow-up of patients during and after cancer therapies. Nevertheless, we need to avoid that patients who survive cancer today develop cardiac dysfunction tomorrow. Therefore such patients should be strictly monitored by both cardiologists and oncologists [134]. In patients with indication for anticancer therapies, a first step would be to evaluate the cardiovascular risk (Table 1). This should be done on the basis of the identification of concomitant cardiovascular diseases and potential cardiovascular complications before anticancer treatments are started, keeping in mind that preexisting hypertension and heart diseases are common in oncologic patients. All in all, clinicians need to recognize and treat cardiovascular risk factors (hypertension, diabetes, current and previous cardiovascular disease, subclinical organ damage previously documented by ECG or echocardiography or carotid ultrasound study, established or subclinical renal disease, age, smoking, dyslipidemia, family history of premature cardiovascular disease, and abdominal obesity) in order to allow long-term continuous therapy with anticancer drugs [1, 45, 57, 67]. Age is indeed an important factor, with elderly patients being at higher risk of both type 1 and type 2 cardiotoxicity [1]. Interestingly, anthracyclines are used for cancer in children, too, and both elderly patients and children can develop LV dysfunction at lower cumulative doses [1, 22, 23]. Indeed the Childhood Cancer Survivor Study showed that, 30 years after anthracyclines, 73% of pediatric cancer survivors would develop at least 1 chronic condition, while 42% would develop a serious life threatening condition or even die of a chronic condition [48, 135]. Greater susceptibility to anthracycline cardiotoxicity has been associated also with female gender [26]. This may be due not only to differences in the pharmacokinetic of anthracyclines between the two sexes, but also because of protection conferred by androgens. Indeed, unpublished data from our laboratory show that testosterone reduces the toxicity of doxorubicin in cultured cardiomyocytes. Finally, it has to be acknowledged that, besides elderly and children, there is a certain risk of cardiotoxicity with occupational exposure to antineoplastic drugs in health care workers, through inhalation of vapors or skin contact with drops. This is particularly true for anthracyclines, while there is no clear evidence of 5-FU cytotoxicity, although there can be chest pain, aspecific ECG disorders, and induction of coronary disease [136].

A complete history and examination, with ECG and blood pressure measurement, are absolutely indicated. Careful monitoring and treatment of blood pressure throughout therapy with angiogenesis inhibitors is important [66, 103]. In such patients, ACE inhibitors, angiotensin receptor blockers (ARBs), and beta-blockers are to be preferred, especially considering that they are effective in preventing HF (Table 1) [67]. The US National Cancer Institute has recently published recommendations to maintain patients' blood pressure at lower than 140/90 mmHg [66, 103].

In spite of the above-mentioned limitations about EF monitoring, Suter and coworkers have proposed an EF based algorithm [1, 137] (EF decreases by 15% points or 10% points to a value below 50) which is easy to follow and can be combined with troponins and BNP. On such basis, when LV dysfunction is detected, systolic function should be reevaluated after 3 weeks, and eventual standard HF treatments can be started [1, 45, 57, 67]. If life expectancy is good, aggressive therapies with devices can also be considered [1, 138]. The priority for oncologic patients is reintroduction of anticancer treatments, even if cardiac therapies are concomitantly administered. Therefore strict monitoring of cardiac function is necessary. At the end of cancer treatments, EF should be monitored to check for late cardiotoxicity 6 months after the conclusion of the therapeutic regimen, then yearly for 2-3 years, and then every 3–5 years for life [1].

## 6. Novel Potential Perspectives in Prevention of LV Dysfunction Induced by Antineoplastic Drugs

According to the 2013 Focused Update of the AHA HF Guidelines, in order to prevent the onset of HF, patients on anticancer drugs should be considered as stage A HF patients [139]. This stage identifies patients at high risk of developing HF, but without structural heart disease or symptoms of HF yet. On such basis, patients on cardiotoxic agents should undergo noninvasive evaluation of LV function with imaging tests and biomarkers (Table 1). HF symptoms and signs should be monitored; cardiovascular risk factors should be addressed. Current strategies to prevent cardiotoxicity (Table 1) include regulation of infusion times to limit peak serum concentrations of anthracyclines, use of liposomal anthracyclines, use of chemotherapy regimens not containing anthracyclines, administering anthracyclines and trastuzumab sequentially rather than concurrently [44, 137, 140, 141], and implementing schemes of cardioprotection (Table 1) [102]. Although the use of preventive cardioprotective therapeutics has been proposed [142–144], most of the studies on HF induced by anticancer drugs have focused on early detection and attenuation or reversion of signs of LV dysfunction [102, 111, 145].

Until now, the vast majority of the studies on cardioprotection have been performed mostly on anthracyclines and, in the case of breast cancer, on anthracyclines + trastuzumab [30, 146] and have been proposing dexrazoxane [147], ACE inhibitors [148], and statins [149, 150] (Table 1). Interestingly, a recent study has evaluated the use of *β*-blockers (Table 1) to prevent anthracycline-induced cardiotoxicity. Concomitant *β*-blocker use may be cardioprotective in patients receiving trastuzumab, anthracyclines, or both [151]. Kalay and colleagues [152] observed that, in patients treated with carvedilol, LV ejection fraction and dimensions do not change with respect to control subject, while undergoing anthracycline chemotherapy. However several preclinical investigations suggest that all *β*-blockers may not be equally effective in preventing chemotherapy-induced cardiotoxicity [102]. Selectivity for *β* receptors seems important for cardiac protection from chemotherapy. In animal models of doxorubicin-induced cardiomyopathy, *β*2 receptor-deficient mice develop severe and lethal acute cardiotoxicity, and the additional deletion of *β*1 receptors rescues this completely [153]. Thus, in animals exposed to anthracyclines, *β*1 activation seems to be cardiotoxic, whereas *β*2 activation is cardioprotective. These data suggest that *β*1 selective antagonist, rather than nonselective *β* blockers, may offer greater protection against anthracycline-induced cardiomyopathy. Molecular mechanisms of cardioprotection from *β*2 receptors activation are activation of prosurvival kinases and decrease in the intracellular concentration of calcium, thus attenuating the mitochondrial dysfunction seen with anthracyclines [154].

Among *β*-blockers, carvedilol also has well-known antioxidant properties [155] and is able to protect cells against doxorubicin toxicity by reducing oxidative stress and apoptosis [156–158]. The same authors [159, 160] also showed the effects of ARBs in preventing oxidative stress and cardiotoxicity from anthracyclines. Nebivolol, a *β*1 selective antagonist and *β*3 agonist, has also been shown to reduce oxidative stress, decrease markers of myocardial injury, and improve LV function [161].

## 7. Conclusions

Cancer drugs currently in use and novel agents that target signaling pathways may all cause problems for the heart. Therefore, to prevent the development of heart failure, it is important that oncologic patients are strictly monitored from cardiologists. Indeed, a fundamental component of cardiooncologic strategies is to establish the vital balance of accepting temporary cardiovascular side effects so as not to impede a patient's ability to benefit from cancer treatment. In a patient with metastatic disease, risk of cardiotoxicity becomes a minor concern; instead, in a patient with a good prognosis, the risk of cardiotoxicity becomes more important [1, 57, 134]. Knowledge of the cardiac effects of anticancer agents balanced with knowledge regarding the natural history of the malignancy and the likelihood of tumor response offers such patients the greatest chance for long-term disease-free survival [1].

In the first place, it is important to recognize patients who are at increased risk for developing cardiac dysfunction associated with cancer treatments. The major mechanisms of left ventricular dysfunction are based on the development of oxidative stress [15, 27–31] and inhibition of cell signaling pathways, by new treatment modalities such as kinase inhibitors, that may also be important for the survival and homeostasis of cardiovascular tissue (Figure 1) [8, 10, 11, 67]. Through observation of side effects caused by new anticancer agents, some cardiovascular signaling pathways have become more clearly understood. Indeed, it is important to understand the relevance of such pathways in order to treat heart failure patients and improve longevity and quality of life for cancer patients. Currently, about 20% of all the investments on drug development is dedicated to small molecule kinase inhibitors, the majority of which (about 80%) being in cancer (with little component in inflammatory and other diseases) [10]. This class is second only to research on drugs targeting G-protein-coupled receptors. Based on the number of kinase inhibitors currently in phase 1 or later clinical trials (about 150 [162]) there appears to be no slowing down in drug development in this area [10]. Beside the fact that this field of research is particularly lucrative, this means that in the next years we are likely going to see a huge increase in the market in the number of compounds which will produce more cardiac dysfunction [10]. In parallel to such increase in drug development, an extremely active field of research is the pursuit of novel strategies to face cardiotoxicity employing new therapeutic approaches or genetic manipulation, miRNAs, and gene transfer [4, 163–172].

## Figures and Tables

**Table 1:** Current insights in prevention, monitoring and treatment of cardiac dysfunction induced by anticancer drugs. Modified from [102].

Prevention	Monitoring	Treatment
*Alternative anticancer strategies* Reduced chemotherapeutic dose Liposomal formulations Less toxic alternatives (epirubicin, lapatinib) *Better patients selection* Age Cardiac risk Cardiac function *Use of cardioprotective drugs* *β*-blockers ACE inhibitors ARBs Dexrazoxane Statins	*Imaging* Assess the best modality Assess the best frequency *Biomarkers* BNP Troponins Novel markers (MPO?)	Hold or stop antineoplastic treatments Start HF therapies
